# Microbial Ecology of European Foul Brood Disease in the Honey Bee (*Apis mellifera*): Towards a Microbiome Understanding of Disease Susceptibility

**DOI:** 10.3390/insects11090555

**Published:** 2020-08-20

**Authors:** Amy S. Floyd, Brendon M. Mott, Patrick Maes, Duan C. Copeland, Quinn S. McFrederick, Kirk E. Anderson

**Affiliations:** 1 Carl Hayden Bee Research Center, USDA Agricultural Research Service, Tucson, AZ 85719, USA; Amy.Floyd@usda.gov (A.S.F.); Brendon.Mott@usda.gov (B.M.M.); 2 Department of Entomology and Center for Insect Science, University of Arizona, Tucson, AZ 85721, USA; pmaes@email.arizona.edu; 3 Department of Microbiology, School of Animal & Comparative Biomedical Sciences, University of Arizona, Tucson, AZ 85721, USA; duancc@email.arizona.edu; 4 Department of Entomology, University of California, Riverside, CA 92521, USA; quinnmc@ucr.edu

**Keywords:** larval microbiome, honey bee microbiome, EFB disease, larvae, *Melissococcus plutonius*, in vitro larval rearing, *Parasaccharibacter apium* strain C6, probiotic

## Abstract

**Simple Summary:**

Honey bees are vital to the agriculture of the world, but like all managed organisms, disease control has become challenging due to the overuse and misuse of antibiotics. Alternate solutions with potential to control disease include natural compounds and probiotic supplements. Probiotic supplements in honey bees have been praised by industry, but studies applying probiotics to honey bee larval disease are lacking and technically challenging. In this study we tested the effectiveness of a demonstrated probiotic (*Parasacharribacter apium* strain C6) to mitigate a damaging larval disease called European Foul Brood (EFB). Based on a controlled laboratory study and two separate trials, the probiotic had no effect on EFB disease. The control groups performed as expected, validating the very sensitive lab procedure used to artificially rear honey bee larvae. Surprisingly, the probiotic provided no survival benefit to larvae in the absence of disease, contradicting past results. We discuss the difficult technique of larval rearing in the laboratory with reference to an improved experimental design introducing disease agents and potential remedies. In summary, our findings indicate that the representation of honey bee health and disease in the laboratory setting requires repeatable validation with reference to rigorous control and natural colony context.

**Abstract:**

European honey bees (*Apis mellifera* Linnaeus) are beneficial insects that provide essential pollination services for agriculture and ecosystems worldwide. Modern commercial beekeeping is plagued by a variety of pathogenic and environmental stressors often confounding attempts to understand colony loss. European foulbrood (EFB) is considered a larval-specific disease whose causative agent, *Melissococcus plutonius*, has received limited attention due to methodological challenges in the field and laboratory. Here, we improve the experimental and informational context of larval disease with the end goal of developing an EFB management strategy. We sequenced the bacterial microbiota associated with larval disease transmission, isolated a variety of *M.* *plutonius* strains, determined their virulence against larvae in vitro, and explored the potential for probiotic treatment of EFB disease. The larval microbiota was a low diversity environment similar to honey, while worker mouthparts and stored pollen contained significantly greater bacterial diversity. Virulence of *M. plutonius* against larvae varied markedly by strain and inoculant concentration. Our chosen probiotic, *Parasaccharibacter apium* strain C6, did not improve larval survival when introduced alone, or in combination with a virulent EFB strain. We discuss the importance of positive and negative controls for in vitro studies of the larval microbiome and disease.

## 1. Introduction

Honey bees are valuable pollinators of agriculture and ecosystems worldwide [1,2]. Recent and sustained colony loss has necessitated a review of pollination ecology and its relation to agriculture [3]. Recent colony loss has been described as multifactorial, involving combinations of environmental stress and disease agents [4,5]. Often associated with colony decline, bacterial diseases of honey bee larvae have become more prevalent worldwide [6]. To confront this growing threat, the patterns and processes that cause or encourage honey bee larval disease are being explored in greater detail [7,8].

The interplay between disease states and the native microbiome is a growing field of study [9,10,11,12,13,14,15]. Protection from pathogens is considered the primary function of the eukaryotic microbiome, and changes to the native honey bee gut microbiota can range from mildly anti-commensal to pathogenic [9,10,15]. Many non-communicable and chronic disease states are associated with microbiome variation, highlighting the importance of microbiome integrity or taxonomic membership in disease susceptibility [10,11,12,13]. In an applied context, bacteria native to the honey bee are being developed as probiotics to provide resistance to pathogens [16]. Species of *Lactobacillus* and *Bifidobacterium* showed an inhibitory effect against larval pathogens [17,18]. Similarly, in *Apis cerana* (Eastern honey bee) *Bacillus subtilis* inhibits a larval pathogen in vitro and when fed to larvae [19]. Another native bacteria, *Parasaccharibacter apium* (strain C6)*,* was also reported to benefit survival when introduced to larvae in vitro [20]. Collectively, these works suggest that the native microbiome can provide solutions to mitigate disease, but the demonstration of biological relevance and colony-level application remains experimentally challenging [16,21].

In the honey bee, disease states and transmission routes have evolved to exploit social context and distinct developmental and reproductive phenotypes [10,22,23]. Honey bee colonies contain a single reproductive queen, developing larvae, functionally sterile workers and stored food; honey and pollen. Queens produce the eggs, but parental care of developing larvae is performed by non-reproductive adult workers called nurse bees; young adults that consume stored pollen, convert it to lipid and protein rich jelly in their hypopharyngeal glands, and secrete this jelly via mouthparts to nourish the queen and developing larvae. Similar to mammalian parental care, these glandular secretions have co-evolved to control microbial growth and support commensal or putatively beneficial bacteria [20,24]. Time from egg to worker pupation is only 7 days, and the larval microbiota is specialized to capitalize on this narrow time window [24]. As larvae develop, they are exposed to a wide variety of commensal and pathogenic microbes vectored from the pollination environment, food stores, nurse worker glands or mouthparts [9,20,25]. Larvae defecate in their wax cells prior to pupation, concurrent with a marked decrease in bacterial load [26]. These same wax cells are cleaned by newly emerged adults that quickly transition into nurse workers and begin to feed developing larvae [27,28].

The two main bacterial diseases of larvae include American Foulbrood (AFB), caused by *Paenibacillus larvae* [4,5], and European Foulbrood (EFB), attributed to *Melissococcus plutonius* [29]. While AFB disease is overt, highly virulent, and caused by a singular bacterial species, the causative factor of EFB and EFB-like symptomology is less well known. With the recent increase and severity of EFB disease worldwide, the last few years has seen great progress elucidating the mechanisms of virulence [8,30,31,32]. Historically, EFB was considered an opportunistic disease, affecting stressed hives. EFB is anecdotally associated with particular crops or environmental conditions and, while economically important, often clears up on its own [33]. Although considered the primary cause of EFB disease, *M. plutonius* often goes undetected when EFB-like symptoms are present. Many other bacteria are detected in association with EFB symptoms, suggesting a disease state associated with opportunistic microbes typically found in the hive environment [8,33]. As a treatment for larval disease, antibiotics damage the native microbiome, select for antibiotic resistance [34], and are now tightly regulated or banned in some countries. Thus, an understanding of the native microbiome may facilitate the development of a long-term EFB management strategy.

A healthy larval microbiota can be dominated by *Lactobacillus* spp. (including *L. kunkeei*) and an Acetobacteraceae referred to as Alpha 2.2, *Parasaccharibacter apium,* more recently renamed *Bombella apis* [20,24,35,36]. Although *Bombella apis* is the scientifically valid nomenclature [36], we use *P. apium* in this manuscript to maintain continuity with past work [20]. *Lactobacillus kunkeei* and *P. apium* reflect a deep ecological and evolutionary history with bee species and the pollination environment in general [24,37] in that particular species and strains are intimately associated with honey bees [38]. Consistently, the growth of flower-derived *Lactobacillus kunkeei* is inhibited by royal jelly, but strains of *L. kunkeei* isolated from honey bee larvae are unaffected [24]. *Parasaccharibacter apium* isolated from honey bee larvae flourished when cultured with royal jelly, but its sister species *Saccharibacter floricola*, also isolated from flowers, is inhibited [24]. It is hypothesized that both of these species have co-evolved with the production of royal jelly, a substance produced only by honey bees [10]. This claim is supported by the prevalence and abundance of these bacterial species in royal jelly, nurse head (hypopharyngeal) glands that produce royal jelly, nurse worker foreguts (a social stomach) and queen guts; a reproductive phenotype fed exclusively royal jelly [10,20,39] (Table 1).

Previous in vitro work suggests that distinct strains of *P. apium* may have varying effects on larval survival, and that *P. apium* strain C6 significantly increased larval survival [20]. In this manuscript, we characterize the niche and ecology of bacteria associated with disease transmission routes using 16S rRNA gene amplicon sequencing of larvae, nurse worker mouthparts, and stored food consumed by nurse workers. Next, we test the putative benefit of *P. apium* strain C6 on developing larvae in vitro by introducing it in a quantitative disease context. We infected honey bee larvae with a pathogenic strain of European Foul Brood (EFB) and determined an in vitro dose response (LD50) for larval survival. We then inoculated EFB infected larvae with the putatively beneficial bacterium; *P. apium* strain C6. We hypothesized that *P. apium* would rescue the larvae from EFB disease; that the survival rate of larvae exposed to both *P. apium* and EFB would increase compared to larvae exposed to only the EFB disease agent.

## 2. Materials and Methods

### 2.1. Characterization of Disease Transmission Routes

To characterize the microbiome associated with larval disease transmission, we examined sources of inoculum associated with the task of feeding larvae. To produce nutritional and antibiotic secretions, adult nurse workers consume stored pollen and honey, then feed larvae via their mouth parts. We examined 16S rRNA gene sequences from honey, stored pollen, nurse mouthparts and larvae (N = 5 libraries per sample type). We extracted DNA from the following substrates from five hives housed at the Carl Hayden Bee Research Center (CHBRC) in Tucson, AZ, USA: Stored pollen, honey, 2nd instar larvae (50 larvae pooled within hive), and nurse bee mouthparts (dissected from 3 nurses pooled within hive) using established techniques [9,20]. Honey samples were obtained by washing ripening, uncapped honey with 100 µL of dH_2_0 in 50 cells per hive. These samples were combined into a 5 mL water/honey sample that was centrifuged at maximum speed for 10 min. The supernatant was removed and the resulting pellet was subjected to DNA extraction [9]. Prior to DNA extraction, all samples were placed into 2 mL bead-beating tubes containing 0.25 g of 0.1-mm silica beads and 1.8 mL of lysis buffer (20 mM Tris-HCl, 2 mM EDTA, 1.2% Triton X-100, pH 8.0). Samples were bead-beaten for 2 min in 30 s intervals, cooled on wet ice for 30 s between each cycle. Samples were then vortexed for 5 s, and the supernatant was removed into 1.5 mL Eppendorf tubes. We then extracted total genomic DNA using a Fermentas Gene Jet Genomic DNA Purification Kit following the protocol for gram-positive bacteria.

#### 2.1.1. PCR and MiSeq

The V1–V2 region of the 16S rRNA gene was amplified using PCR primers 27F (AGRGTTTGATCMTGGCTCAG) and 338R (AGTGCTGCCTCCCGTAGGAGT). Amplification was performed using the HotStarTaq Plus Master Mix Kit (Qiagen, USA) under the following conditions: 94 °C for 3 min, followed by 28 cycles of 94 °C for 30 s, 53 °C for 40 s and 72 °C for 1 min, with a final elongation step at 72 °C for 5 min. After amplification, PCR products were checked in 2% agarose gel to determine the success of amplification and the relative intensity of bands. Samples were purified using calibrated Ampure XP beads. Purified PCR product was used to prepare DNA library following Illumina TruSeq DNA library preparation protocol. Sequencing was performed at MR DNA (www.mrdnalab.com, Shallowater, TX, USA) on a MiSeq following the manufacturer’s guidelines.

#### 2.1.2. MiSeq Sequence Analysis

Sequences were processed using MOTHUR v.1.35.1 [47]. Forward and reverse reads were joined using the make.contigs command. After the reads were joined the first and last five bases pairs were removed using the SED command in unix. Sequences were then screened, using the screen.seqs command, to remove any sequences containing ambiguous bases. Unique sequences were generated using the unique.seqs command. A count file containing group information was generated using the count.seqs command. Sequences were aligned to Silva SSUREF database v102 [48] using the align.seqs command. Sequences not overlapping in the same region and columns not containing data were removed using the filter.seqs command. Sequences were pre-clustered using the pre.culster command. Chimeras were removed using UCHIME [49] and any sequences that were not of known bacterial origin were removed using the remove.seqs command. All remaining sequences were classified using the classify.seqs command. All sequences with only one or two (single/doubletons) associated reads were removed using the AWK command in unix. A distance matrix was constructed for the aligned sequences using the dist.seqs command. Sequences were classified with the RDP Naive Bayesian Classifier [50] using a manually constructed training set containing sequences sourced from the greengenes 16S rRNA database (version gg_13_5_99 accessed May 2013 [51], the RDP version 9 training set, and all full length honeybee-associated gut microbiota listed in NCBI. Operational taxonomic units (OTUs) were generated using the cluster command. Representative sequences for each OTU were generated using the get.oturep command. To further confirm taxonomy, resulting representative sequences were subject to a BLAST query using the NCBI nucleotide database.

To better define the niche of *P. apium*, we compared the average abundance of *P. apium* in honey, stored pollen, larvae, and nurse mouthparts to the abundances of *P. apium* observed previously for other related honey bee niches including royal jelly, the glands that produce royal jelly (hypopharygeal glands), gut compartments of queens and worker castes; cell cleaners, nurses and foragers [20,28,40,41,42,43,44,45,46].

#### 2.1.3. Sequence Data Deposition

All sequence data were deposited in GenBank under Sequence Read Archive (SRA) accession PRJNA640829.

### 2.2. General Bacterial Culturing

To identify a virulent strain of EFB and acquire isolates associated with EFB disease, we cultured bacteria from larvae associated with EFB disease acquired from the Beltsville Bee Research Lab collection, and an outbreak of EFB diagnosed near Crestone, Colorado during the summer of 2018. Larvae were collected into sterile 2 mL tubes and shipped on ice to the Carl Hayden Bee Research Center in Tucson, AZ, USA. Saline (500 µL) was added to the tube and the larvae were homogenized with a cotton swab. This aqueous mixture was streaked onto plates containing KSBHI or Medium 1 from Arai et al. [52]. Plates were incubated for 7 days at 34 °C under anaerobic conditions using anaerobic jars, gaspacks, and anaerobic indicator strips. Resulting isolates were picked and banked, PCR amplified and Sanger sequenced at the 16S rRNA gene as described previously [9].

From the Carl Hayden Bee Research Center microbe collection, we selected *Parasaccharibacter apium* strain C6 as a putative rescue bacterium. *P. apium* strain C6 is a honey bee-specific strain of Acetobacteraceae considered beneficial to larval survival [20], and contains genes associated with bacterial competition and survival in the insect gut [20,53]. We cultured strain C6 according to Corby-Harris et al. [20].

### 2.3. Bacterial Inhibition Assay

Royal and worker jelly are highly antimicrobial, but bacteria co-evolved with the honey bee can flourish in royal jelly and the in vitro larval diet [20,24]. We tested the ability of our two experimental strains to survive in commercially available royal jelly. We plated both *M. plutonius* and *P. apium* with varying concentrations of the same royal jelly used in the rescue experiment, from full strength to highly dilute (diluted with sterile deionized water). Each bacterium was plated in their respective media type. We cultured *M. plutonius* strain AF5 in KSBHI, and *P. apium* strain C6 in SDA [20]. Filter paper discs were saturated with royal jelly of different dilutions (100%, 50% and 10%) and placed onto the inoculated plates. A disc was placed in each quarter of the plate. The assay was performed in triplicate resulting in 12 reps for each bacterium and dilution.

While *P. apium* strain C6 grows rapidly in both royal jelly and the larval diet [20], the effects of the larval diet on *M. plutonius* growth and survival are unknown. We verified the survival of *M. plutonius* (strain AF5) in the larval diet by culturing a time series on KSBHI media. We first cultured the physiological saline solution containing the *M. plutonius* in triplicate to verify initial inoculant concentrations (time 0). We then cultured the inoculated larval diet at 6, 12, 24, 48, and 72 h to detail bacterial growth and survival.

### 2.4. Rearing of Host (A. mellifera) Larvae In Vitro

We used in vitro larval rearing to identify a virulent strain of EFB bacteria that reliably killed honey bee larvae. We then determined the lethal dose required to kill approximately 50% of the larvae (LD50). Using specially designed grafting tools, newly hatched larvae were removed from the wax frames of 10 healthy robust colonies, and floated spiracle side up into 2 mL wells of sterile 24-well plates typically used for in vitro larval rearing. The larval diet consisted of royal jelly, yeast exact, fructose, glucose and water according to [54]. Larvae were incubated at 34 °C and 90% humidity for 6 days. On day 6, prior to pupation, larvae were moved to a petri dish with filter paper to defecate, then returned to the incubator at 34 °C and 80% humidity. After 24 h the larvae were moved back into a clean 24-well plate to pupate and emerge in the incubator.

### 2.5. LD50 Determination M. plutonius

*Melissococcus plutonius* virulence varies by strain [30,31,32], and is often detected in asymptomatic hives. Culturing *M. plutonius* as defined by cell morphology and 16S sequence does not guarantee strain virulence [29,52,55]. We therefore tested the virulence of multiple *M. plutonius* isolates by inoculating larvae in vitro. We determined an LD50 for the chosen *M. plutonius* strain (AF5), by introducing an inoculant dilution series to larvae. A scrape of 7-day-old growth on KSBHI agar was mixed into 1000 µL of saline. An arbitrary 10% dilution was equivalent to 64 CFU/µL in the diet. From this initial concentration, an order of magnitude serial dilution was prepared ranging from 64 to 0.0064 CFUs/µL. To define an LD50, each inoculant concentration was fed in 225 µL of diet (see below) to 24 larvae in vitro. After 7 days growth we determined CFU (colony forming unit) counts for each concentration as the average of three plates. We used a spectrophotometer and optical density (OD) at 600 nm to construct a standard curve for CFU counts, and then relied on OD600 readings to determine *M. plutonius* concentrations.

### 2.6. In Vitro Rescue Experiment

To test whether *P. apium* strain-C6 [20] was able to rescue larvae infected with *M. plutonius,* we fed both *M. plutonius* and *P. apium* to larvae in vitro. We used the quantified *M. plutonius* (strain AF5) as the disease agent in the *P. apium* rescue experiment. We introduced as independent variables; (1) the lowest *M. plutonius* concentration that killed 100% of the larvae (6.4 CFU/µL, positive control), (2) the *M. plutonius* concentration that killed 50% of the larvae (0.64 CFU/µL a treatment concentration most likely to reveal rescue potential), and (3) no *M. plutonius* bacteria (a negative control). To each of these 3 conditions we added the same concentration of *P. apium* strain C6 used in past experiments demonstrating a benefit (3 CFUs/µL; [20]). A total of 288 larvae were assayed, 48 for each of the six experimental conditions. We ran two independent trials with 24 larvae per condition per trial. Larvae were fed all diet and inoculant a single time point, as first instars. We distributed each of the six treatment conditions equally among the plates to remove the potential for a “plate effect”.

The larval diet was comprised of 34 mL sterile distilled water, 6 g of D-glucose (6%), 6 g of D-fructose, 1 g yeast extract and 53 g fresh commercially available RJ from GloryBee [54]. The negative control contained no bacteria and was comprised only of the above ingredients and saline solution as a control for bacterial introduction. We used the OD600 standard curve to estimate inoculant concentration, resulting in a final concentration of 6.4 CFU/µL and 0.64 CFU/µL in the diet. For the three treatments containing *P. apium* (C6), glycerol stock was grown up on SDA agar plates for 48 h at 34C. Bacteria was then scraped into saline and plated in ten-fold dilutions onto SDA agar plates. CFUs were counted to determine concentration of the bacteria in saline. A 60 CFUs/µL dilution was added to the diet resulting in a final concentration of 3 CFUs/µL of *P. apium,* the same concentration used for previous in vitro larval rearing trials [20].

#### Statistical Analysis—Mortality

We assessed total larval mortality at the end of the seven-day period using a chi-square contingency table to test for treatment differences. P-values were adjusted based on a multiple comparison false discovery rate adjustment (FDR).

## 3. Results

### 3.1. Niches Associated with Disease Transmission

We sequenced the 16S rRNA gene from various niches implicated in disease transmission. Next-generation sequencing returned 1,777,744 quality trimmed reads (300 bp) from 25 amplicon libraries (summarized in Supplementary Material, Table S1). Beebread was represented by 314,705 reads averaging 31,470 per library, honey by 630,010 reads averaging 126,002 per library, mouthparts by 502,473 reads averaging 100,495 per library and larvae by 330,556 reads averaging 66,111per library.

Across all libraries, a total of 2285 OTUs were resolved at 97% similarity. Average library coverage was high across all samples. Good coverage was 0.99 (±0.004) for the beebread samples, 1.00 (±0.0003) for mouthpart samples, 1.00 (±0.0003) for honey samples and 1.00 (±0.0001) for the larval samples. We used the inverse Simpson’s diversity index to portray niche diversity (larger vales indicate greater diversity) resulting in a 7.98 (±7.17) for beebread, 6.04 (±5.51) for mouthparts, 2.27 (±1.32) for honey and 2.15 (±0.47) for larvae (Supplementary Material, Table S2).

**Figure 1:**
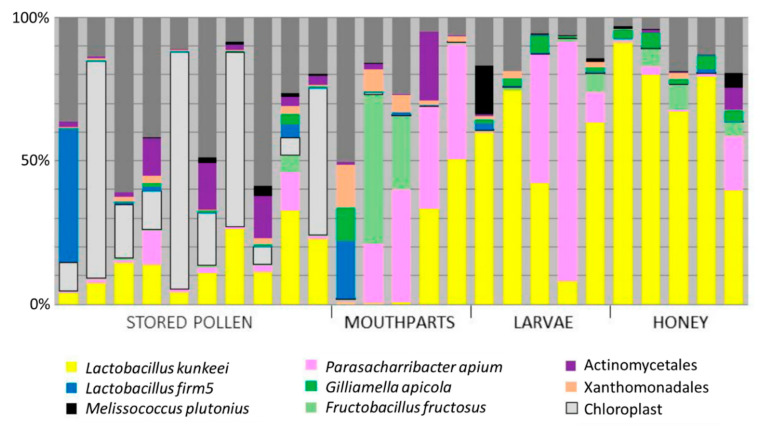
Bacterial communities associated with potential hive refugia (food storage) and transmission from adult mouthparts to larvae. We sequenced the 16S rRNA gene from four niches associated with larval feeding (see methods). Colors represent various bacterial species, genera or broad groups. The chosen primer set (V1–V3 region) also amplifies chloroplast genes from pollen (light grey). Dark grey represents the relative proportion of bacterial groups not listed in the figure legend (see Table S1).

Sequences corresponding to the EFB disease agent, *Melissococcus plutonius*, were detected in all 25 libraries, but had the greatest relative abundance in honey and larvae, and the least relative abundance on nurse worker mouthparts (Figure 1). The number of sequence reads designated as *M. plutonius* is significantly associated with total sampling effort across all libraries (Pearson’s R = 0.45, *n* = 25, *p* = 0.02).

Also detected in all libraries, *P. apium* comprised 12.6% of the total sequences obtained from nurse mouthparts, honey, and second instar larvae (Table 1). The larval libraries were 25% *P. apium* on average, the honey libraries 4%, and the mouthparts 22%. Results from the current survey and previous publications indicate that *P. apium* is abundant (>20% relative abundance) in larvae, nurse worker mouthparts, worker foreguts (social stomach), nurse worker hypopharyngeal glands, royal jelly, and queen guts, and less abundant in adult worker guts and honey rich food stores (Table 1).

### 3.2. Bacterial Culturing

Based on BLASTn (Supplementary Material, Table S3), the bacteria recovered by our culturing effort were most similar to *Melissococcus plutonius* DAT 561 (*n* = 49), a variety of *Enterococcus* species (*n* = 22), primarily *Enterococcus faecalis* (*n* = 15), *Lactobacillus kunkeei* (*n* = 16), four strains of *Paenibacillus alvei* (*n* = 12), primarily *Paenibacillus alvei* strain Oa17A (*n* = 9). The remaining sequences were *Bifidobacterium* spp. (*n* = 1), and *Parasaccharibacter apium* strain G7_7_3c (*n* = 1). The EFB strain chosen to challenge larvae in vitro was named Mp5, and originated from an outbreak in Colorado in 2018.

### 3.3. Bacterial Inhibition Assay

To verify that *M. plutonius* strain AF5 survives in the larval environment, we cultured it in the presence of royal jelly and the in vitro diet. Both *M. plutonius and P. apium* grew in their preferred culture media in the presence of royal jelly. We observed no inhibition zone around royal jelly-containing discs, and no growth inhibition for either bacterium at royal jelly concentrations from dilute (10%) to concentrate (100%). *P. apium* strain C6 is known to proliferate (increase by a factor of 5) in the larval diet [20]. When *M. plutonius* was inoculated into the larval diet, it decreased quickly but survived at very low concentration for 72 h (Table 2).

### 3.4. LD50 Determination M. plutonius

**Figure 2:**
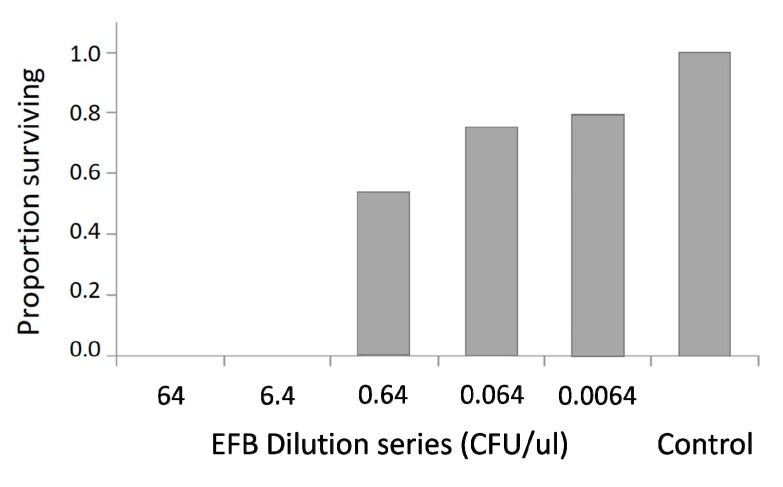
Survival of larvae exposed to a dilution series of *M. plutonius* strain AF5. Larvae were inoculated in vitro (N = 24 for each concentration) and survival was recorded at Day 6. The control received diet and saline with no bacteria. We designated the 6.4 CFU/µL dilution as LD100, and the 0.64 CFU/µL dilution as LD50 for use as positive controls in the in vitro rescue assay.

We tested four strains of *M. plutonius* for virulence against larvae in vitro, and found that three of the four resulted in larval mortality. We chose one of these virulent strains (AF5) to determine an LD50 value and perform further experiments. Consistent with expectations, in vitro larval survival decreased with increasing concentration of *M. plutonius* strain AF5. (Figure 2). Following infection, *M. plutonius* dilutions were plated in triplicate to confirm CFUs introduced to larvae in the diet.

### 3.5. In Vitro Rescue Experiment

**Figure 3:**
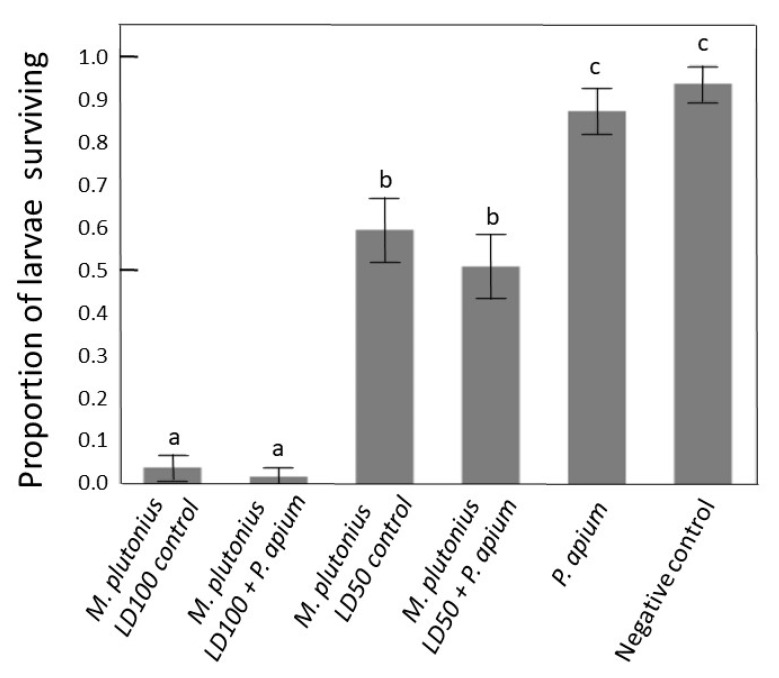
Proportion of larvae surviving *M. plutonius* challenge and *P. apium* rescue. Error bars represent two independent trials (see methods). From Figure 1, LD100 represents (6.4 CFU/µL) of *M. plutonius* AF5, and LD50 is (0.64 CFU/µL) killing approximately 50% of the larvae by 6 days of age. *P. apium* was introduced at 3 CFU/µL, the concentration used in Corby Harris et al. [20]. Columns with the same letter do not differ for larval survival.

The negative control produced >90% survival in both rescue trials validating the in vitro rearing procedure (Figure 3). The two positive controls also produced larval survival at expected proportions; low survival in the high *M. plutonius* concentration and survival not significantly different from 50% for the LD50 *M. plutonius* concentration. We introduced *P. apium* strain C6 as a rescue bacterium for EFB infection. Treatment groups inoculated with both *P. apium* strain C6 and a high concentration of *M. plutonius* strain AF5 did not differ, indicating no effect of *P. apium* on larval survival. The same was true for strain C6 and the lower concentration of strain AF5. The treatment group inoculated with only strain C6 did not differ significantly from the negative control group in either trial (Figure 3).

## 4. Discussion

Honey bee foul brood diseases have serious economic consequences for beekeeping and pollination services globally [56,57]. Although various assays can detect larval pathogens in hives [56,58] there is much to be learned about the ecology and epidemiology of *M. plutonius,* the causative agent of European Foul Brood (EFB) disease. Based on broad sampling throughout the United Kingdom, *M. plutonius* is comprised of at least three genetically distinct groups [7] and virulence differs markedly by strain [30,31,32]. *Melissococcus plutonius* is often found in the guts of workers so may be easily transmitted among hives when they are placed in proximity [13]. Recent increases in antibiotic resistance and incidence of EFB and EFB-like brood disease have spurred efforts to understand virulence including genetic sequencing, comparative genomic analysis, the contribution of secondary bacteria and targeted molecular biology [8,30,31,32,52,55,59,60,61,62]. Understanding the microbial ecology associated with *M. plutonius* will provide a functional context of disease progression and suggest potential solutions to mitigate disease. Here, we expand on past work to describe and discuss the general context of EFB disease transmission in hives, identify virulent strains and biologically relevant inoculant concentrations of *M. plutonius*, and test whether a putatively beneficial bacterium (*P. apium* strain C6) can rescue or diminish EFB related larval mortality.

### 4.1. Context of Disease Transmission

Results from our next-generation 16S rRNA amplicon survey suggests that the prevalence and abundance of *M. plutonius* is diminished in niches with higher bacterial diversity (beebread and mouthparts; Figure 1). *Melissococcus plutonius* is relatively abundant when abiotic factors are extreme (e.g., low pH, high acidity, low water availability), as is the case with 2nd instar larvae and the tops of ripening honey cells (Figure 1). That *M. plutonius* might best survive in extreme environments with low competition is consistent with recently described gene sets [60]. It is hypothesized that 2nd instar larvae are most vulnerable to EFB infection, concurrent with the presumed diet transition from royal to worker jelly. Our results agree with culture-based assays [24] that 1st and 2nd instar larvae can be dominated by *P. apium* or *L. kunkeei,* both fast-growing bacteria often considered at least commensal if not beneficial [20]. Both of these species groups are comprised of multiple strains that possess functional differences and occupy a variety of honey bee niches [10]. Given the genetic variation within each species group, it is likely that function varies by niche and strain. Consistent with our findings and the literature, *P. apium* is clearly co-evolved with royal and worker jelly, and considering the totality of available data, *P. apium* proliferates in a wide variety of in-hive niches seemingly defined by the social network of food sharing and the potential to encounter royal jelly (Table 1).

Based on spatial occurrence patterns, *M. plutonius* may be somewhat ubiquitous in disease free hives. It was represented in every niche and library with relative abundance correlated significantly with sequencing effort (Figure 1, Table S1). The relatively low representation of *M. plutonius* on the nurse bee mouthparts may reflect an oxygenated niche and/or highly competitive environment. Also considered ubiquitous in past investigations [29,57] the putative secondary invaders *Paenibacillus alvei* and *Enterococcus faecalis* were detected at very low prevalence and abundance throughout our survey, despite the deep sequencing effort (Table 2). We detected multiple strains of *P. alvei*, but based on 16S gene sequences, *Enterococcus* spp. were poorly defined taxonomically, with variability likely inflated by the chosen 16S gene region. Perhaps as suggested, *P. alvei* is a saprophyte on dead larvae [8] and may only be detected in abundance during a disease outbreak.

### 4.2. Bacterial Competition In Vitro

When cultured in their preferred growth media, neither *P. apium* strain C6 nor *M. plutonius* strain AF5 were inhibited by royal jelly. When *P. apium* strain C6 was added to the in vitro rearing diet, it increased by a factor of five [20], but results from the present experiment show that *M. plutonius* strain AF5 was diminished by the larval diet, but it was still detected after 72 h (Table 2). These contrasting results may highlight virulence associated factors that vary among *M. plutonius* strains and/or larval microbial ecology that may influence disease progression. Strain AF5 likely belongs to clade CC12, because it is highly virulent [30], exhibits similar growth requirements [59,60,61,62], and shares 99.5% 16S rRNA gene sequence similarity with *M. plutonius* strain DAT561, a member of the CC12 clade [7] (Supplemental information Table S3). Although *M. plutonius* strain AF5 may not survive for long in the environment of the larval diet, even small numbers can successfully find their preferred niche in the larval gut, multiply and cause disease (Figure 2). Consistent with this hypothesis, the introduction in larval diet of relatively small doses of *M. plutonius* strain AF5 still produced significant larval mortality relative to the negative control (Figure 3).

The early growth patterns of *M. plutonius* and competing bacteria that typically colonize newly hatched larvae may be important factors in the progression and treatment of EFB disease. Results presented here and in the literature indicate that *M. plutonius* has evolved in strong niche proximity with *P. apium* (Figure 1). Because *P. apium* (strain C6) has been reported to increase larval survival [20], we hypothesized that it may provide benefits in a disease context, protecting larvae from the establishment of *M. plutonius.* However, our results show that *P. apium* strain C6 does not rescue larvae from the effects of EFB disease associated with *M. plutonius* strain AF5 (Figure 3). Larvae inoculated with both *M. plutonius* and *P. apium* strain C6 did not survive significantly better than larvae inoculated with only *M. plutonius*. Moreover, the in vitro procedure was validated by the consistent performance of both positive and negative control groups (Figure 3). We speculate that *M. plutonius* avoids competition with *P. apium* strain C6 in the larval gut via niche partitioning or competitively inhibition. Future studies would benefit by using MLST genotyping to characterize *M. plutinious* strains [60], varying the introduction times and concentrations of probiotic and pathogen, and quantifying the proliferation and establishment of *M. plutonius* and potential probiotics. To place our sequencing results (Figure 1) in a broader context, our microbiome survey revealed >30 OTUs classified as *P. apium*, many abundant in larval guts (Supplementary Materials, Table S1). A single *P. apium* OTU accounted for 85% of all *P. apium* in larvae, but the OTU matching *P. apium* strain C6 accounted for less than 1% of all *P. apium* in larvae, suggesting that *P. apium* strain C6 is not a dominant larval strain. As detailed earlier (Table 1), various *P. apium* strains occupy many niches throughout the hive and colony. Testing more strains and more combinations of strains will better reveal the potential of the native larval microbiome to mitigate disease.

### 4.3. Larval Assay In Vitro

Our in vitro results do not agree with our past study [20]. In the present study, we found that negative control survival (no introduced bacteria) did not differ from survival of larvae inoculated with *P. apium* strain C6 (Figure 3). Thus, in contrast to our 2014 study, we found no effect of *P. apium* strain C6 on larval survival in the absence of disease. Our continued lab experience suggests that differences between the studies were a consequence of the in vitro rearing technique. In our 2014 study, the low and variable nature of control and treatment survival between trials within the study suggested a lack of reproducibility as discussed by the authors [20]. More specifically, negative control survival was exceedingly low, and differed significantly between the two trials at 40 and 70 percent survival. However, the statistical model in [20] did not consider trial variation, thus, it was difficult to conclude whether treatment effects or trial variation was the greatest contributor to the statistical differences reported in [20].

In the present study, we used the same in vitro larval diet containing the same bacterial strain (C6) and inoculum concentration used in the 2014 study. However, in 2014, the diet and inoculate was introduced multiple times during larval development; uneaten diet and inoculate were removed with a pipette at each daily feeding interval, and replaced with fresh diet and inoculate [20]. While daily feeding more closely replicates the continuous nature of feeding in the colony, our continued lab experience suggests that multiple disturbances over time can influence larval survival in a plate-specific manner, increasing assay error. Specifically, in the 2014 study, (1) we segregated treatments and controls by plate, allowing for a “plate-specific effect” on survival, and (2) we disturbed plates multiple times for feedings and inoculations (repeated exposure), increasing the probability of larval contamination, damage or drowning. In contrast, the methods of the present study limit these random factors by, (1) mixing treatments across plates to minimize plate-specific effects and, (2) minimally disturbing larvae (single exposure) to reduce the opportunity for contamination, damage or drowning.

In the honey bee system, larval rearing in vitro has been continuously refined to approximate what occurs in colonies under natural conditions; high and repeatable larval survival [54,63,64,65]. Accordingly, an a priori threshold for high and repeatable survival of the negative control (Figure 2) drove the present experimental design. In-hive larval survival under natural conditions is generally greater than 90% [64,65], and represents the established standard for in vitro rearing. If negative control survival in vitro falls below this natural threshold, it indicates a failure of the in vitro rearing protocol or larvae sourced from an unhealthy colony. Given a priori survival expectations in the absence of a positive control, a survival benefit attributed to an introduced treatment could only attain statistical relevance if survival of the negative control falls significantly below that of the putatively beneficial treatment. In turn, the beneficial treatment would be difficult, if not impossible to distinguish from repeatable in vitro rearing technique. In other words, when recording survival as the only dependent variable, the demonstration of a benefit would rely on poor survival of the negative control, an inherent design flaw. Given the established expectation of high and repeatable survival in the negative control, introducing a factor that is hypothesized to benefit larval survival requires a positive control for adequate design.

## 5. Conclusions

The results and methodology presented here contribute to the development of a microbiome-centric model of health and disease in honey bees. Assessing the microbial ecology of larvae including *Melissococcus plutonius* and associated larval microbiomes will promote a better understanding of the infection process and suggest potential management strategies beyond broad-spectrum antibiotics. Within the context of an improved experimental design, *P. apium* strain C6 was commensal, producing no effect on larval survival. Our results highlight the importance of both positive and negative controls when designing ecological studies involving disease agents and potential remedies. Because we performed our experiments under controlled lab conditions, we exposed larvae to less variety and abundance of naturally occurring hive microbiota and fresh royal jelly components. For future in vitro experiments, the ability to classify native vs. non-native microbiota and represent the associated microbiome and social dynamics during larval development will be critical for modeling larval health and disease in the natural hive setting.

## Figures and Tables

**Table 1:** Relative abundance of *Parasaccharibacter apium* by niche and study.

Study	Site	Tissue or Niche	Developmental Stage or Caste	Total Sequences	% *P. apium*
**Relative abundance of *P. apium* from whole worker gut samples**					
Martinson et al. 2012 [40]	AZ	whole guts	9-day-old nurses (N = 3)	78,595	0
Martinson et al. 2012 [40]	AZ	whole guts	30-day-old forager (N = 1)	17,910	0
Sabree et al. 2012 [41]	MA	whole guts	12 days old	106,344	1
Moran et al. 2012 [42]	AZ, MD	whole guts	in hive, outer frames	329,550	1
Corby-Harris et al. 2014a [43]	AZ	whole guts	pollen foragers	354,505	6
Anderson et al. 2016 [28]	AZ	whole guts	3-day-old bees ^A^	90,893	0
Anderson et al. 2016 [28]	AZ	whole guts	7-day-old bees ^A^	166,113	0
**Relative abundance of *P. apium* from honey-rich food storage environments**					
Anderson et al. 2014 [44]	AZ	corbicular pollen	forager corbiculae	85,043	6
Anderson et al. 2014 [44]	AZ	stored pollen	hive environment	116,593	3
present study	AZ	ripening honey	hive environment	784,635	4
present study	AZ	stored pollen	hive environment	314,705	6
**Relative abundance of *P. apium* from niches with >20% *P. apium* abundance**					
Corby-Harris et al. 2014a [43]	AZ	crop (foregut)	pollen forager	195,264	43
Corby-Harris et al. 2014a [43]	AZ	crop (foregut)	nurse	113,405	32
Corby-Harris et al. 2014b [20]	AZ	nurse headglands	nurse	127,157	34
Corby-Harris et al. 2014b [20]	AZ	royal jelly	worker head glands	105,820	40
Kapheim et al. 2015 [45]	IL	dissected guts	queen (N = 4)	78,355	38
Tarpy et al. 2015 [46]	NC	dissected guts	queen ^B^	5000	24
Anderson et al. 2018 [39]	AZ, CA	mouthparts	queen ^C^	1,305,332	89
Anderson et al. 2018 [39]	AZ, CA	midgut	queen ^C^	1,572,752	86
Anderson et al. 2018 [39]	AZ, CA	ileum	queen ^C^	1,419,416	38
present study	AZ	mouthparts	nurse worker	502,473	22
present study	AZ	whole body	worker larvae 2nd instar	330,556	25

^A^ Only bees fed a natural diet are included. ^B^ Libraries were subsampled to 5000. ^C^ Corrected for rRNA copy number and community size.

**Table 2:** *M. plutonius* strain AF5 decreases in the in vitro larval rearing diet.

Time	* CFU 1	CFU 2	CFU 3	Avg	CFU/µL in Diet
0 h	285	261	253	266	33.29
6 h	114	142	152	136	13.6
24 h	91	74	57	74	1.48
48 h	59	85	53	66	1.31
72 h	5	9	7	7	0.14

* CFU colony forming units, plated in triplicate.

## Supplementary Materials

The following are available online at https://www.mdpi.com/2075-4450/11/9/555/s1, Table S1. Microbiome raw read count (relative abundance) by niche and taxonomy; Table S2. Alpha diversity of microbiome samples by niche; Table S3. Results culturing diseased larvae from various locales experiencing EFB outbreaks.
